# How Many Conformations of Enzymes Should Be Sampled for DFT/MM Calculations? A Case Study of Fluoroacetate Dehalogenase

**DOI:** 10.3390/ijms17081372

**Published:** 2016-08-20

**Authors:** Yanwei Li, Ruiming Zhang, Likai Du, Qingzhu Zhang, Wenxing Wang

**Affiliations:** 1 Environment Research Institute, Shandong University, Jinan 250100, China; lyw@sdu.edu.cn (Y.L.); zrm0118@163.com (R.Z.); wxwang@sdu.edu.cn (W.W.); 2 Hubei Key Laboratory of Agricultural Bioinformatics, College of Informatics, Huazhong Agricultural University, Wuhan 430070, China.; dulikai@mail.hzau.edu.cn

**Keywords:** quantum mechanics/molecular mechanics, enzymatic dehalogenation, Boltzmann-weighted average barrier, disproportionate effect

## Abstract

The quantum mechanics/molecular mechanics (QM/MM) method (e.g., density functional theory (DFT)/MM) is important in elucidating enzymatic mechanisms. It is indispensable to study “multiple” conformations of enzymes to get unbiased energetic and structural results. One challenging problem, however, is to determine the minimum number of conformations for DFT/MM calculations. Here, we propose two convergence criteria, namely the Boltzmann-weighted average barrier and the disproportionate effect, to tentatively address this issue. The criteria were tested by defluorination reaction catalyzed by fluoroacetate dehalogenase. The results suggest that at least 20 conformations of enzymatic residues are required for convergence using DFT/MM calculations. We also tested the correlation of energy barriers between small QM regions and big QM regions. A roughly positive correlation was found. This kind of correlation has not been reported in the literature. The correlation inspires us to propose a protocol for more efficient sampling. This saves 50% of the computational cost in our current case.

## 1. Introduction

The quantum mechanics/molecular mechanics (QM/MM) method is a powerful tool to model biochemical processes. It can probe enzymatic mechanisms at the atomic level which is generally hard to be experimentally explored [1,2,3,4,5]. Ever since the pioneer study by Warshel and Levitt on a realistic enzyme system in 1976 [6], the QM/MM technique has been improved and widely applied. Currently, there are generally two ways to calculate the features (e.g., structural parameters, energies) of enzymatic reactions. One is “dynamic sampling” (e.g., the umbrella sampling method) [7,8,9]. Combined with QM/MM, dynamic sampling gives a relatively accurate description of the dynamic behavior of an enzyme. This method, however, is highly computationally inefficient and is in most cases applicable with only low-level QM methods (e.g., PM3) [10]. Alternatively, adiabatic mapping was proposed. This method samples a random enzymatic conformation of enzyme residues using molecular dynamics (MD), and locates the reaction pathway in this conformation (the enzyme-substrate-water complex). Although high-level QM methods (e.g., density functional theory (DFT), or even ab initio) can be used in adiabatic mapping, this method likely generates biased results [11,12].

To address this dilemma, adiabatic calculations using DFT/MM in enzymes with multiple sampled conformations have been widely reported [13,14,15,16,17,18]. Reaction pathways are generally optimized in each of the sampled conformations. The average energy barrier is computed by averaging all the barriers from multiple conformations. Two averaging methods, the direct arithmetic average and the Bolzmann-weighted average (also known as the exponential average), have been proposed [19,20,21]. Recently Kästner and coworkers have tested the two averaging methods. They concluded that the Bolzmann-weighted average better reproduces the experimental value than the direct arithmetic average method [22]. Although the advantage of the Bolzmann-weighted average method has been proposed for 20 years [23], it was only recently applied in studying enzymatic reactions.

A challenging problem, however, is how to determine the minimum number of enzymatic conformations required for the convergence of the averaged energy barrier. Different articles have tentatively proposed different numbers of conformations, considering 3–10 [21] and 5–10 [24] conformations have been recommended, and three [13,14,15], four [16], five [17], six [18,19], seven [20], eight [21], 10 [25], 20 [26], or even 40 [27] conformations have been recently used. The justification, however, is supposed to be provided for the number of conformations being used. An effective strategy is to manually pick conformations from MD that already resemble the transition state geometry [22]. However, this strategy has two limitations. First, the “transition-state-like” is conceptually ambiguous. Identification of important structural parameters could be arbitrary. For instance, only after analyzing results from 20 conformations, an angle was found to be more sensitive toward the energy barrier than all the other tested structural parameters for fluoroacetate dehalogenase catalyzed processes [28]. Second, this “manually picking” strategy is not applicable to the cases in which the first step of a multi-step enzymatic process is not rate-determining.

**Scheme 1:**
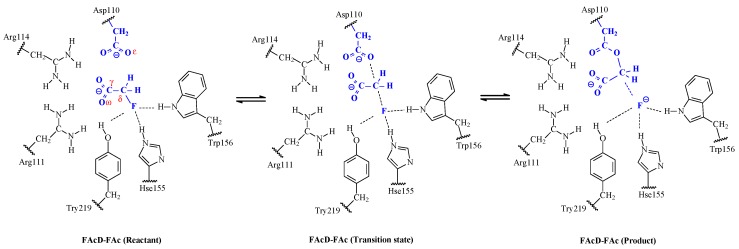
The dehalogenation process catalyzed by fluoroacetate dehalogenase. The atoms in small quantum mechanics (QM) regions are indicated in bold blue while atoms considered in big QM regions are indicated in black. The boundaries between the QM and molecular mechanics (MM) regions are indicated by wavy lines.

Here, we provide a strategy to determine the minimum number of conformations required for the convergence of energies in enzymatic reactions. Defluorination and dechlorination reactions catalyzed by fluoroacetate dehalogenase (FAcD) [29,30] were selected as representative. The mechanism of the reaction has been well demonstrated. As shown in Scheme 1, the reaction is triggered by residue Asp110 attacking the C_δ_ atom of the substrate fluoroacetate, which leads to the cleavage of the C–F bond. Using the strategy proposed in the present study, about 50% and 30% computational costs were saved for the defluorination and dechlorination processes. The results will be valuable in guiding the work-flow of future DFT/MM studies and enhance its application in modeling enzymatic chemical processes.

## 2. Results and Discussion

We studied the defluorination of FAc, and the dechlorination of FAc catalyzed by FAcD. Based on the substrate and size of the QM region used, four systems are generated. They are FAcD-FAc-S, FAcD-FAc-B, FAcD-ClAc-S, and FAcD-ClAc-B. Here “FAc” and “ClAc” refer to the substrates fluoroacetate and chloroacetate while “S” and “B” refer to the small or big QM regions used during DFT/MM calculations.

### 2.1. Convergence Criteria

**Figure 1:**
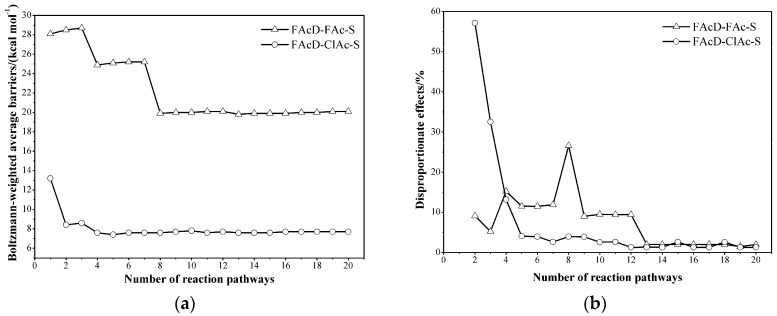
The convergence of (**a**) Boltzmann-weighted average barrier and (**b**) disproportionate effect of systems FAcD-FAc-S and FAcD-ClAc-S in 20 conformations.

Currently it is still challenging to determine the number of conformations that are supposed to be sampled for converging the averaged energy barrier of a given enzyme system. To address this issue, two convergence criteria were proposed. The first criterion is the convergence of the Boltzmann-weighted average barrier. The Bolzmann-weighted average method has been shown to be an excellent approach for analyzing the fluctuations of energy barriers in multiple conformations compared to the direct arithmetic average method [21,22]:

(1) $$\Delta E=-RT\;\text{ln}\{\frac{1}{n}\sum_{i=1}^{n}\text{exp}\left(\frac{-\Delta E_{i}}{RT}\right)\}$$

where Δ*E* is the average barrier, *n* is the total number of sampled conformations, *ΔE_i_* is the energy barrier of conformation *i*. The second criterion is the convergence of the disproportionate effect. For a small *n*, if the set of starting conformations includes one conformation with an anomalously low energy barrier, this will have a dramatic disproportionate effect (*DE*) on the Boltzmann-weighted average barrier [28]:

(2) $$DE=\frac{\Delta E^{a-l}-\Delta E^{a}}{\Delta E^{a}}\times 100\%$$

where Δ*E^a−l^* is the Boltzmann-weighted average barrier calculated by neglecting the conformation with the lowest energy barrier, Δ*E^a^* is the Boltzmann-weighted average barrier with all the conformations considered. *DE* is another important indicator for determining whether the sampled number of conformations is sufficient. To decrease the number of conformations needed, the Boltzmann-weighted average barrier and disproportionate effect were considered as “converged” when the corresponding numerical changes are less than 5% in at least five consecutive iterations. This can prevent the results from being converged to an incorrect value. Figure 1 shows the Boltzmann-weighted average barrier versus the number of sampled conformations for FAcD-FAc-S and FAcD-ClAc-S. The Boltzmann-weighted average barrier is converged after 13 and nine conformations (Figure 1a) while the disproportionate effect is converged after 18 and 17 conformations (Figure 1b) for the systems FAcD-FAc-S and FAcD-ClAc-S. However, taking both of the convergence criteria into consideration, 18 and 17 are the minimum number of conformations that should be sampled for studying the systems FAcD-FAc-S and FAcD-ClAc-S. 

### 2.2. Small QM Region versus Big QM Region

As shown in Figure 1a, the Boltzmann-weighted average barrier for defluorination (20.1 kcal·mol^−1^) is much higher than dechlorination (7.7 kcal·mol^−1^), which is inconsistent with the experimental observation that FAcD favors defluorination over dechlorination [31]. This is presumably because some key residues of the strong interaction with the substrates were not included in the QM region in FAcD-FAc-S and FAcD-ClAc-S. To test our assumption, FAcD-FAc-B and FAcD-ClAc-B were calculated with residues His155, Trp156, and Tyr219 in the QM region. The Boltzmann-weighted average barriers for defluorination and dechlorination are then 11.4 and 14.5 kcal·mol^−1^, respectively, which are consistent with the experiment [28]. This shows the necessity of the test on the QM boundary. This has also been found by many other studies [32,33].

**Figure 2:**
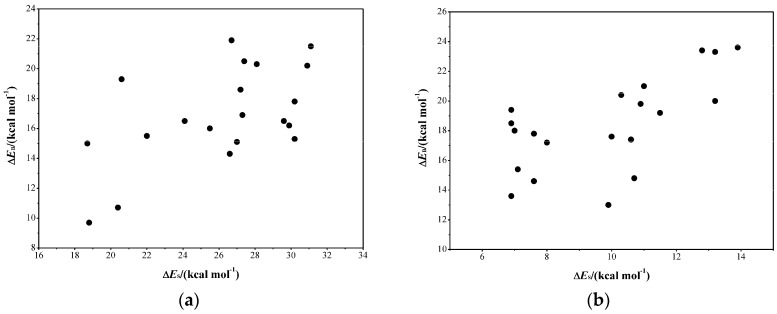
The correlation of energy barriers between systems (**a**) FAcD-FAc-S and FAcD-FAc-B and systems (**b**) FAcD-ClAc-S and FAcD-ClAc-B.

What we are interested in here is whether there is a correlation of energy barriers between small and big QM region systems. The correlation is helpful for the qualitative prediction of the energy barriers for big QM region systems (accurate, computationally inefficient) based on small QM region calculations (inaccurate, computationally efficient). As shown in Figure 2a (FAcD-FAc-S/FAcD-FAc-B) and Figure 2b (FAcD-ClAc-S/FAcD-ClAc-B), a positive relationship between energy barriers in small and big QM systems was found by analyzing results from 20 independent conformations. The positive relationship suggests that if the energy barrier of a typical conformation with a small QM region were low, the corresponding energy barrier with a large QM region is likely low. Take the system FAcD-FAc-S as an example; the lowest energy barrier among 20 studied conformations is 18.7 kcal·mol^−1^ (conformation sampled at 6.5 ns), while the energy barrier of the same conformation in the system FAcD-FAc-B is 15.0 kcal·mol^−1^, the fourth lowest among 20 studied conformations (details are shown in Table S1).

Based on the correlation, a protocol for efficient adiabatic DFT/MM calculations is proposed. Firstly, pre-screen the enzymatic reactions with the minimum number of QM atoms. The number of conformations can be determined by checking the convergence of the Boltzmann-weighted average barrier and the disproportionate effect; Secondly, align the obtained barriers from lowest to highest. Thirdly, choose the lowest energy barrier and calculate the corresponding energy barrier with the big QM region considered; Finally, successively choose more conformations based on the alignment and calculate the corresponding energy barriers (big QM region calculations) until the convergence of the Boltzmann-weighted average barrier and the disproportionate effect are reached. It should be noted that the correlation between energy barriers in small and big QM systems will not influence the reliability of the protocol proposed. Worse correlation generally means more computational cost: more conformations (big QM region calculations) are needed before the criteria are reached.

### 2.3. Determine the Minimum Number of Conformations to Be Sampled

**Figure 3:**
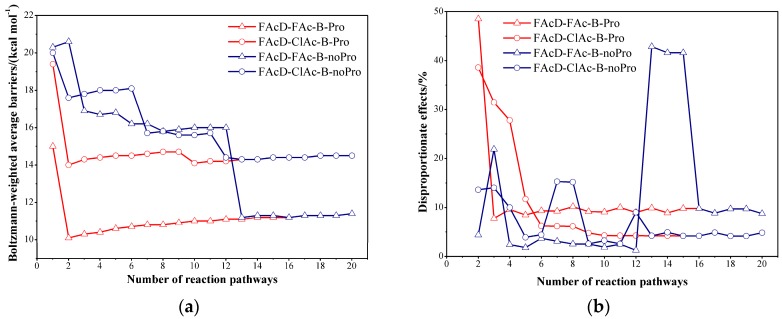
The convergence of (**a**) Boltzmann-weighted average barrier and (**b**) disproportionate effect of systems with protocols (FAcD-FAc-B-Pro and FAcD-ClAc-B-Pro) considered, and systems without protocols considered (FAcD-FAc-B-noPro and FAcD-ClAc-B-noPro).

The convergence of the Boltzmann-weighted average barrier and the disproportionate effect for systems with a big QM region (FAcD-FAc-B and FAcD-ClAc-B) were shown in Figure 3. Two cases were tested: (1) use the protocol (FAcD-FAc-B-Pro and FAcD-ClAc-B-Pro); (2) do not use the protocol, randomly increase the number of conformations and test the convergence (FAcD-FAc-B and FAcD-ClAc-B). According to two proposed convergence criteria, the systems FAcD-FAc-B-Pro and FAcD-ClAc-B-Pro (protocol used) are converged after eight and 11 conformations, which indicates that the energy barrier correlation between the systems FAcD-FAc-S and FAcD-FAc-B is slightly better than the correlation between the systems FAcD-ClAc-S and FAcD-ClAc-B. The systems FAcD-FAc-B and FAcD-ClAc-B (protocol not used) are only converged after 20 and 18 conformations. It is worth mentioning that the converged results from using the protocol reached the same accuracy by directly calculating all the 20 configurations (big QM region calculations). Combining the fact that the demanded computational resource for small QM region calculations only accounts for 10% of that demanded for big QM region calculations in the present study, applying the protocol will approximately save 50% and 30% computational cost for defluorination and dechlorination processes catalyzed by FAcD.

## 3. Materials and Methods

The dehalogenation of substrates fluoroacetate (FAc) and chloroacetate (ClAc) by fluoroacetate dehalogenase (FAcD) were selected as two representatives in this study. The QM/MM protocol used here is similar to our previous study [28] and only important details of the methods will be briefly summarized. The QM/MM calculations were performed by using ChemShell [34,35] platform, which integrates programs Turbomole [36] and DL-POLY [37]. The geometries of the intermediates and transition states were optimized by using hybrid delocalized internal coordinates optimizer and microiterative transition state (TS) optimizer under the B3LYP/6-31G(d,p)//CHARMM22 level [38]. Frequency calculations were performed to verify the one imaginary frequency character of transition state structures, and the suitability of the transition vector was also confirmed. In pre-screen, QM atoms were treated by B3LYP/6-31G(d,p) method while MM atoms were treated by CHARMM22 force field. All QM atoms and MM atoms within 20 Å of element F or Cl were allowed to move while the other MM atoms were fixed during QM/MM calculations. The QM region (14 atoms) used for pre-screening in the present study only contain residues Asp110 and substrate (FAc or ClAc) rather than relatively bigger QM region (90 atoms) described in our previous study [28]. Twenty conformations were extracted from 10 ns molecular dynamics simulation with 0.5 ns interval. They were used for the QM/MM calculations for both defluorination and dechlorination processes. This strategy may avoid biased sampling to some extent and has been extensively applied in many other studies [17,20,27].

## 4. Conclusions

The convergence of the Boltzmann-weighted average barrier and the disproportionate effect are used here to determine the number of conformations that are sufficient to converge the energetics of defluorination (18 for FAcD-FAc-S, 20 for FAcD-FAc-B) and dechlorination (17 for FAcD-ClAc-S, 18 for FAcD-ClAc-B) catalyzed by fluoroacetate dehalogenase. A protocol on the basis of the roughly positive correlation between energy barriers of small and big QM regions was proposed. The protocol saves about 50% and 30% computational cost for defluorination and dechlorination in the present study.

## Supplementary Materials

Supplementary materials can be found at www.mdpi.com/1422-0067/17/8/1372/s1.
