# Supplementary material for: How Many Conformations of Enzymes Should Be Sampled for DFT/MM Calculations? A Case Study of Fluoroacetate Dehalogenase
Source: Int J Mol Sci. 2016 Aug 20;17(8):1372. doi: 10.3390/ijms17081372 (PMC5000767; doi:10.3390/ijms17081372)
Supplement: Supplementary file 1 [file ijms-17-01372-s001.pdf]

Supplementary Materials: How Many Conformations of Enzymes Should Be Sampled for DFT/MM Calculations? A Case Study of Fluoroacetate Dehalogenase

Yanwei Li, Ruiming Zhang, Likai Du, Qingzhu Zhang and Wenxing Wang

Table S1. Twenty calculated energy barriers for systems FAcD-FAc-S, FAcD-FAc-B, FAcD-ClAc-S, and FAcD-ClAc-B. The unit is in kcal·mol^{-1}.

Snapshots/ns	FAcD-FAc-S	FAcD-FAc-B	FAcD-ClAc-S	FAcD-ClAc-B
0.5	26.6	14.3	7.1	15.4
1	27.2	18.6	7.6	17.8
1.5	27.0	15.1	10.3	20.4
2	30.9	20.2	10.0	17.6
2.5	20.4	10.7	11.5	19.2
3	27.4	20.5	10.6	17.4
3.5	25.5	16.0	10.9	19.8
4	18.8	9.7	6.9	13.6
4.5	22.0	15.5	9.9	13.0
5	30.2	17.8	6.9	18.5
5.5	26.7	21.9	13.2	23.3
6	20.6	19.3	10.7	14.8
6.5	18.7	15.0	13.9	23.6
7	29.6	16.5	7.6	14.6
7.5	30.2	15.3	12.8	23.4
8	27.3	16.9	7.0	18.0
8.5	24.1	16.5	6.9	19.4
9	29.9	16.2	11.0	21.0
9.5	31.1	21.5	8.0	17.2
10	28.1	20.3	13.2	20.0
